# Rapamycin protects kidney against ischemia reperfusion injury through recruitment of NKT cells

**DOI:** 10.1186/s12967-014-0224-z

**Published:** 2014-08-19

**Authors:** Chao Zhang, Long Zheng, Long Li, Lingyan Wang, Liping Li, Shang Huang, Chenli Gu, Lexi Zhang, Cheng Yang, Tongyu Zhu, Ruiming Rong

**Affiliations:** Department of Urology, Zhongshan Hospital, Fudan University, Shanghai, China; Shanghai Key Laboratory of Organ Transplantation, 180 Fenglin Road, Shanghai, 200032 China; Biomedical Research Center, Zhongshan Hospital, Fudan University, Shanghai, 200032 China; Department of Transfusion, Zhongshan Hospital, Fudan University, 180 Fenglin Road, Shanghai, 200032 China; Qingpu Branch Zhongshan Hospital, Fudan University, 1158 Gongyuan Road East, Shanghai, 201700 China

**Keywords:** Rapamycin, Renal ischemia reperfusion injury, NKT cell, Chemokine

## Abstract

**Background:**

NKT cells play a protective role in ischemia reperfusion (IR) injury, of which the trafficking in the body and recruitment in injured organs can be influenced by immunosuppressive therapy. Therefore, we investigated the effects of rapamycin on kidneys exposed to IR injury in early stage and on trafficking of NKT cells in a murine model.

**Material and methods:**

Balb/c mice were subjected to kidney 30 min ischemia followed by 24 h reperfusion. Rapamycin (2.5 ml/kg) was administered by gavage daily, starting 1 day before the operation. Renal function and histological changes were assessed. The proportion of NKT cells in peripheral blood, spleen and kidney was detected by flow cytometry. The chemokines and corresponding receptor involved in NKT cell trafficking were determined by RT-PCR and flow cytometry respectively.

**Results:**

Rapamycin significantly improved renal function and ameliorated histological injury. In rapamycin-treated group, the proportion of NKT cells in spleen was significantly decreased but increased in peripheral blood and kidney. In addition, the CXCR3^+^ NKT cell in the kidney increased remarkably in the rapamycin-treated group. The chemokines, CXCL9 and CXCL10, as the ligands of CXCR3, were also increased in the rapamycin-treated kidney.

**Conclusions:**

Rapamycin may recruit NKT cells from spleen to the IR-induced kidney to ameliorate renal IR injury in the early stage.

## Introduction

Ischemia-reperfusion (IR) injury, an inevitable impairment during renal transplant surgery, remains one of the major causes of acute kidney injury and represents the most prominent factor leading to delayed graft function after transplantation [1].

Immunosuppressive therapy is necessary for patients receiving organ transplantation, but it affects the resolution of tissue damage induced by IR injury [2]. Recent years, it has seen a heated argument focusing on the effect of rapamycin, a kind of mTOR inhibitor, on ischemic acute kidney injury. Many studies indicated a damage-promoting role of rapamycin during IR injury through such mechanisms as increase of NF-κB activity [3], pro-apoptosis [4], promotion of renal oxidative/nitrosative stress [5] and aggravation of tissue infiltration of leukocytes [6]. There was also evidence, however, indicating that rapamycin inhibited apoptosis by preventing phosphorylation of pro-apoptotic protein such as p53 and activation of mitochondrial cell death pathway [7]. In addition, rapamycin enabled to enrich CD4^+^CD25^+^Foxp3^+^ regulatory T (Treg) cells to exert anti-inflammatory effects during IR process [8]. Our preliminary work showed that rapamycin attenuated renal IR injury at the early stage (1d after IR) but aggravated it at the late stage (7d after IR).

Natural killer T (NKT) cells, expressing both T cell receptors (TCRs) an natural killer cell receptors, have been thought as a bridge between the innate and adaptive immune systems [9]. Specific to the murine model, NKT cells express both the surface marker of T cells—CD3 and that of NK cells—NK1.1, which can be used to identify NKT cells in murine tissues. NKT cells can migrate in response to ligands for the inflammatory chemokine receptors CXCR3 (CXCL9 and CXCL10) and CXCR6 (CXCL16), and regulatory chemokine receptors CCR7 (secondary lymphoid-tissue chemokine (SLC) and CCL21), CXCR4 (stromal cell-derived factor-1 and CXCL12), and CXCR5 (B cell-attracting chemokine-1 and CXCL13); but not to ligands for other chemokine receptors [10]. NKT cells have recently been reported to regulate autoimmune disease and allogeneic immune response in according animal models [11,12]. Notably, relevant research demonstrated a significant protective role for NKT cells in acute kidney injury caused by ischemia reperfusion. Modulation of cellular infiltration and cytokine expressions along with molecular changes such as hypoxia-inducible factor-1α and IL-10 are the main mechanisms for protection [13].

Thus, on the basis of the knowledge of the comprehensive regulatory effects of rapamycin on immune system and a significant renal protective function of NKT cells, we hypothesized that rapamycin may protect IR-induced kidney injury at the early stage through recruiting NKT cells from periphery to the injured site.

## Materials and methods

### Materials

Rapamycin oral solution (East China Pharmaceutical, Co Ltd., Hangzhou, China) was diluted in normal saline (1:10) for gastric gavage.

### Renal ischemia reperfusion injury model

Male BALB/c mice (weighing 20-25 g), were obtained from Shanghai Slac Lab Animal, Co., Ltd, and bred in an experimental animal room of SPF grade. All animal procedures were performed according to the guidelines of the Care and Use of the Laboratory Animal Ethical Commission of Fudan University. Mice were randomly divided into three groups (n = 5): (1) Sham group; (2) IR group: IR injury with 0.5 ml normal saline gavage; (3) Rapa group: IR injury with 0.5 ml diluted Rapamycin suspension gavage at 24 h, 1 h before operation, as well as 12 h after operation. For the renal IR model, the mice were anaesthetized with pentobarbital at a dosage of 0.1 g/kg body weight intraperitoneally. Core body temperature was maintained at 37°C. The abdominal cavity was exposed via a midline incision, both kidneys were exposed and the renal pedicles were carefully isolated. Bilateral renal occlusion for 30 min was performed using non-traumatic vascular clamps. Occlusion was confirmed by observing blanching of the entire kidney surface. After removing the renal clips, the kidneys were observed for a further 5 min to ensure color change indicating blood reperfusion. Afterwards, 0.5 ml saline at 37°C was injected into the abdomen and the incision was sutured in two layers [14].

Animals were ethically sacrificed at 24 h after IR injury and the whole blood drawn from the heart was centrifuged at 4°C, 3000 rpm, for 25 min to obtain the serum sample. The level of serum creatinine (Scr) and blood urea nitrogen (BUN) was measured by the automatic biochemistry analyzer (Hitachi 7060, Hitachi Ltd., Tokyo, Japan). One kidney was harvested and transversally cut at the midline. One half was fixed with 10% buffered formalin for histological assessment, while the rest was frozen at −80°C for western blot and RT-PCR. The other kidney and the spleen were kept in phosphate-buffered saline (PBS) in preparation for flow cytometry analysis.

### Single cell suspension preparation and flow cytometry

Spleen and kidney tissue were harvested from mice at given time points and crushed in mesh bags to obtain single cell suspensions, in which RBC were lysed with hypotonic erythrocyte lysis buffer (TIANGEN Biotech, Beijing, China). Thereafter all single cells were re-suspended in staining buffer (BD Bioscience, San Diego, CA, USA). The following monoclonal antibodies (1 mg/ml; BD PharMingen) were used to identify NKT cells: FITC-labeled anti-mouse CD3 (Clone: 17A2) and APC-labeled anti-mouse NK1.1 (Clone: PK136). CXCR3 expression was determined by PE-labeled anti-mouse CXCR3 (CD183) (Clone: CXCR3-173). Flow cytometry data were acquired using BD FACS Aria II (BD Biosciences) and analyzed with FlowJo software 6.0 (Tree Star Inc., Ashland, OR, USA).

### Quantitative Real-time PCR

Total RNA was extracted from renal tissues with Trizol reagent (Invitrogen, Carlsbad, USA). One μg of total RNA was reverse transcribed into cDNA using a RevertAid™ First Strand cDNA Synthesis Kit (Fermentas, Glen Burnie, USA). Real-time quantitative PCR (QPCR) was performed using the SYBR *Premix Ex Taq* Kit (Takara Bio Inc., Otsu, Japan) in the ABI Prism 7900HT system (Applied Biosystems, Foster City, CA, USA). Thermocycler conditions included 2-minute incubation at 50°C, then 95°C for 10 minutes; this was followed by a 2-step PCR program, as follows: 95°C for 15 seconds and 60°C for 60 seconds for 40 cycles. GAPDH was used as an internal control to normalize differences in the amount of total RNA in each sample. Primers are listed in Table 1

**Table 1:** **The sequences of the primers**

**Gene name**		**Primer sequence**
CXCL9	Sense	TCC TTTTGGGCATCATCTTC
	Antisense	TTCCCCCTCTTTTGCTTTTT
CXCL10	Sense	ACTGCATCCATATCGATGAC
	Antisense	TTCATCGTGGCAATGATCTC
CXCL12	Sense	TGCATTTATAGCATACGGTATGA
	Antisense	GCGTTAATAAGGATTGCCATTT
CXCL13	Sense	GAGGCAGATGGAACTTGAGC
	Antisense	CTGGGGATCTTCGAATGCTA
CXCL16	Sense	CTGACTCAGCCAGGCAATGG
	Antisense	TGAGTGGACTGCAAGGTGGA
CCL21	Sense	CCAAGTTTAGGCTGTCCCATC
	Antisense	GGGCTACTGGGCTATCCTCT
GAPDH	Sense	TATGAGGAACCGCATCGCTG
	Antisense	TAGCATGAGTTGGCACCCACTG

.

### Western blot

Twenty μg protein from kidney homogenate were separated on 15% (wt/vol) poly acrylamide denaturing gels and electro-blotted onto Hybond-C membranes. These membranes were blocked with 5% (wt/vol) milk, separately probed with anti-S6RP (Cell Signaling Technology, Boston, USA) and anti-p-S6RP (Cell Signaling Technology). For the loading control, the same membranes were probed with anti-β-actin antibody (1:10,000 dilution, Abcam, Cambridge, UK), then incubated with peroxidase-conjugated secondary antibodies (1:10,000 dilution, Jackson ImmunoResearch, West Grove, USA) at room temperature for 1 h. Immunoreactive bands were visualized using ECL substrate (Thermo Fisher Scientific, Rockford, USA) and a Bio-Image Analysis System (Cell Biosciences, Inc., Santa Clara, USA). The semi-quantitative analysis results were expressed as optical volume density (OD × mm^2^) and normalized by β-actin for loading (AlphaView Software 3.3, Cell Biosciences, Inc.).

### Histological assessment

Renal specimens were fixed in 10% neutral buffered formalin and paraffin-embedded. Deparaffinized sections (5–10 μm) were stained with hematoxylin and eosin (HE). The tissue sections were blind-labeled and reviewed by two renal pathologists. A histologic score system was used to estimate the renal damage, which was graded by the percentage of tubule injury: 0 (<1%); 1 (1–10%); 2 (11–20%); 3 (21–40%); 4 (41–60%); 5 (61–75%); 6 (>75%) [15]. The scores represented the severity of tubular injury (including loss of proximal tubule brush border, cell swelling or vacuolization, and cell necrosis): the score ranges of 1–2 represented mild injury, 3–4 represented moderate injury, and 5–6 represented severe injury.

### *In situ* end-labeling apoptotic cells

Five micrometer paraffin sections were used to label fragmented DNAs *in situ* with digoxigenin-deoxyuridine (dUTP) by terminal deoxynucleotidyl transferase (TdT) using a TUNEL Apoptosis Detection Kit (Millipore, MA, USA) [16,17]. Briefly, sections were digested by 40 μg/ml proteinase K (EMD Chemicals, NJ, USA) for 15 min at 37°C, incubated with TdT and digoxigenin-dUTP at 37°C for 60 min and transferred to wash/stop buffer for 30 min. After adding anti-digoxigenin-peroxidase complex for 30 min, these sections were developed by DAB substrate. Apoptotic cells were examined at 400× magnification over 20 fields for semi-quantitation.

### Statistical analyses

Statistical analysis of the data was performed with the two-tailed independent t-test between two groups, using SPSS 19.0 software (SPSS Inc, Armonk, NY, USA). Values of P less than 0.01 were considered significant. All values were presented as mean ± SD.

## Results

### Rapamycin attenuated renal dysfunction, ameliorated renal histologic damage and apoptosis

**Figure 1:**
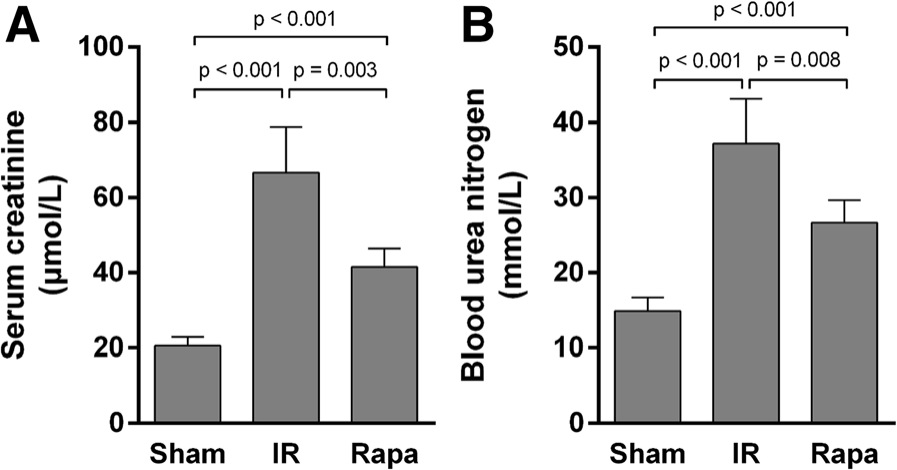
**Renal function.** Serum creatinine was markedly increased in IR group compared with Sham group, but after rapamycin treatment, serum creatinine was significantly reduced **(A)**. Urea nitrogen level showed the similar trend as serum creatinine **(B)**. Data are expressed as mean ± SD (n = 5).

Serum creatinine and blood urine nitrogen were markedly increased by IR injury compared with sham group. After rapamycin treatment, Scr and BUN level were significantly reduced compared with the IR group (Figure 1).

**Figure 2:**
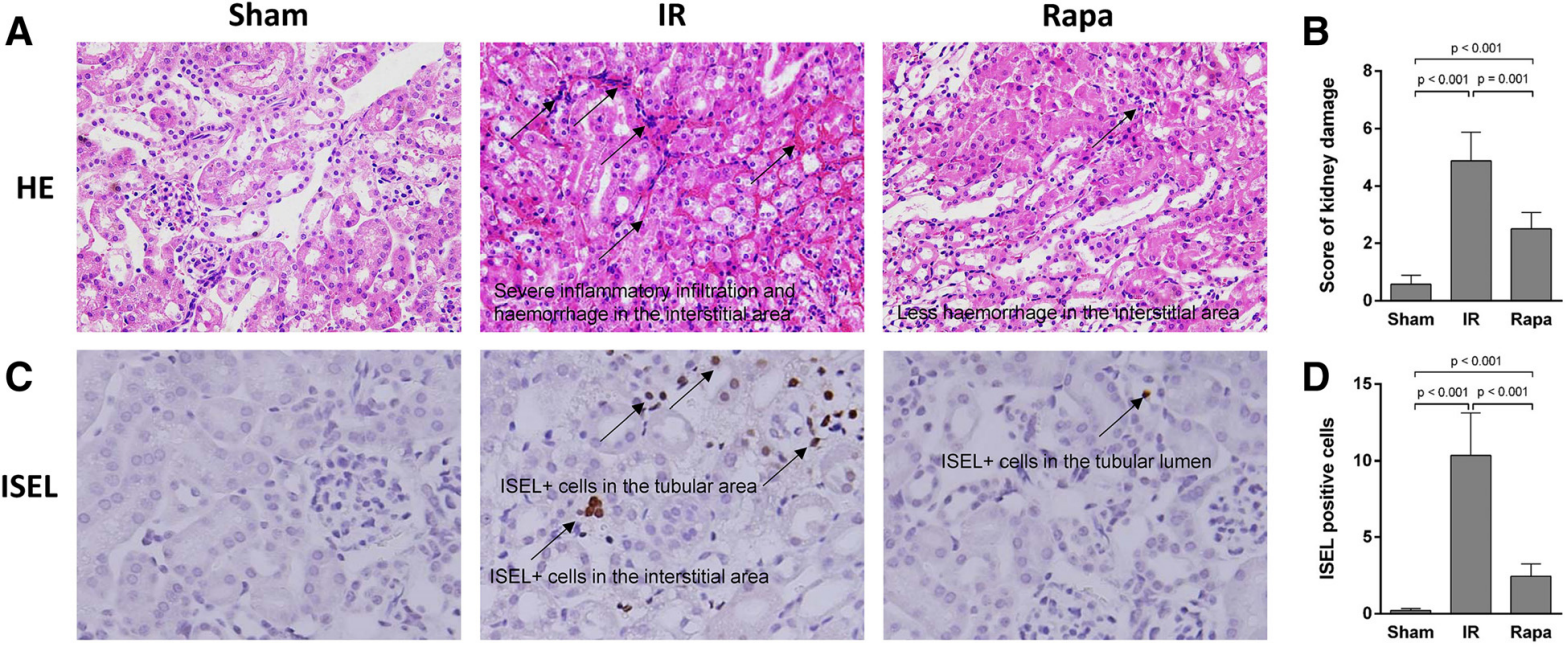
**Renal tissue damage and apoptosis.** Tubular vacuolation, protein casts, severe inflammatory infiltration as well as haemorrhage were seen in the IR group. In contrast, less tissue damage was seen after rapamycin treatment **(A)**. The score of kidney damage assessed in HE sections at 400× magnification showed that Rapamycin significantly ameliorated kidney damage compared with IR group **(B)**. Apoptotic cells were detected by ISEL fragmented DNAs at 400× magnification. They were hardly seen sham group, but increased after IR injury, which were markedly decreased after Rapamycin treatment **(C)**. The number of apoptotic cells was remarkably higher in IR group than in Rapa group **(D)**. Data are expressed as mean ± SD (n = 5).

Histological assessment in each group was performed in HE stained sections. There were significant tubular changes including loss of brush border, dilation of renal tubules, as well as inflammatory infiltration and hemorrhage in the interstitial area cells following IR injury compared with the sham group. In contrast, rapamycin treatment significantly ameliorated tubular lesions (Figure 2A). The semi-quantitative assessment of histologic lesion showed a significantly lower score in the rapamycin group compared with the IR group post 24 h reperfusion (Figure 2B).Consistently, the number of apoptotic cells, detected by *in situ* end-labeling (ISEL) fragmented DNAs, in tubulointerstitial areas (Figure 2C) was significantly decreased by rapamycin compared with the IR group (Figure 2D).

### Rapamycin recruited NKT cells to the IR-injured kidney

**Figure 3:**
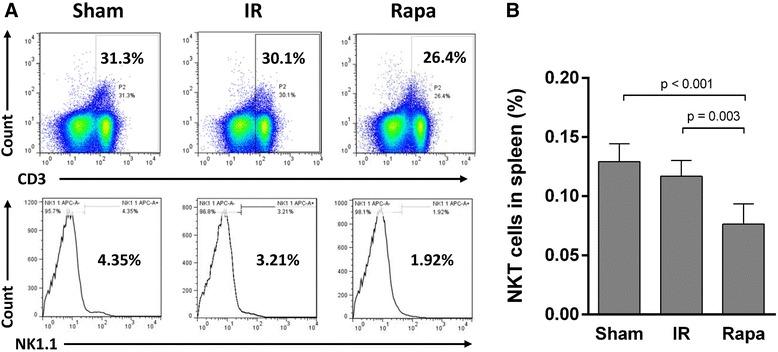
**The proportion of NKT cells in spleen.** Lymphocytes were gated from all RBC-free single cell suspensions by FSC and SSC (not showed), then CD3^+^ cells were gated in all lymphocytes, and NK1.1^+^ cells were selected in CD3^+^ lymphocytes. The proportion of NKT cells (CD3^+^NK1.1^+^) in the spleen was calculated as the proportion of CD3^+^ cells in all lymphocytes multiplied by the NK1.1^+^ cells in CD3^+^ lymphocytes as the total quantity of splenocytes was manipulated to equal for each test **(A)**. The proportion of NKT cells (CD3^+^NK1.1^+^) in spleen was significantly lower in Rapa group than in IR group **(B)**. Data are expressed as mean ± SD (n = 5).

**Figure 4:**
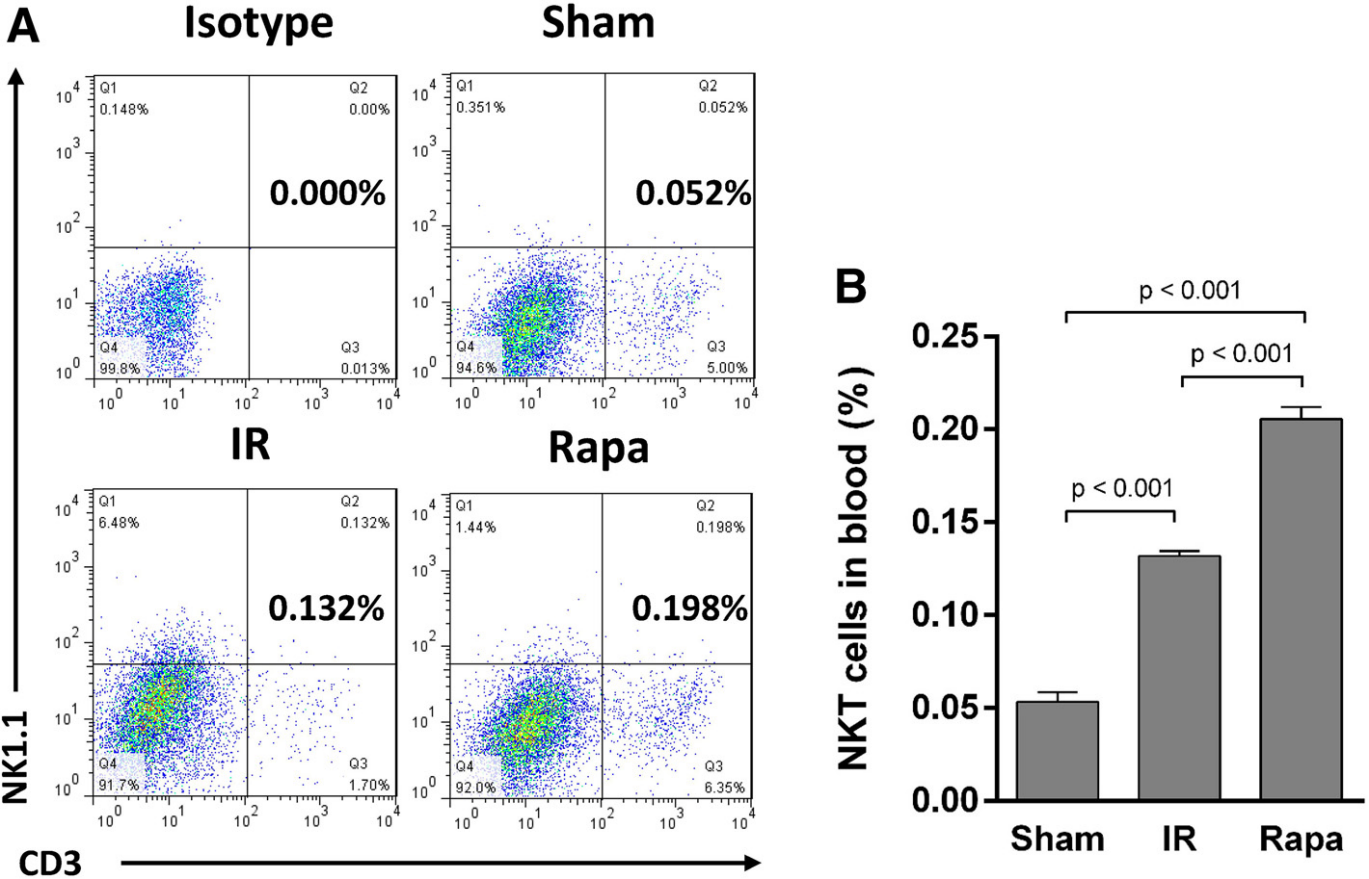
**The proportion of NKT cells in peripheral blood.** Lymphocytes were gated from all RBC-free single cell suspensions by FSC and SSC, and CD3^+^NK1.1^+^ cells were selected in all lymphocytes. Isotype group was performed as control **(A)**. After Rapamycin treatment, the proportion of NKT cells (CD3^+^NK1.1^+^) was significantly increased compared with IR group and Sham group **(B)**. Data are expressed as mean ± SD (n = 5).

**Figure 5:**
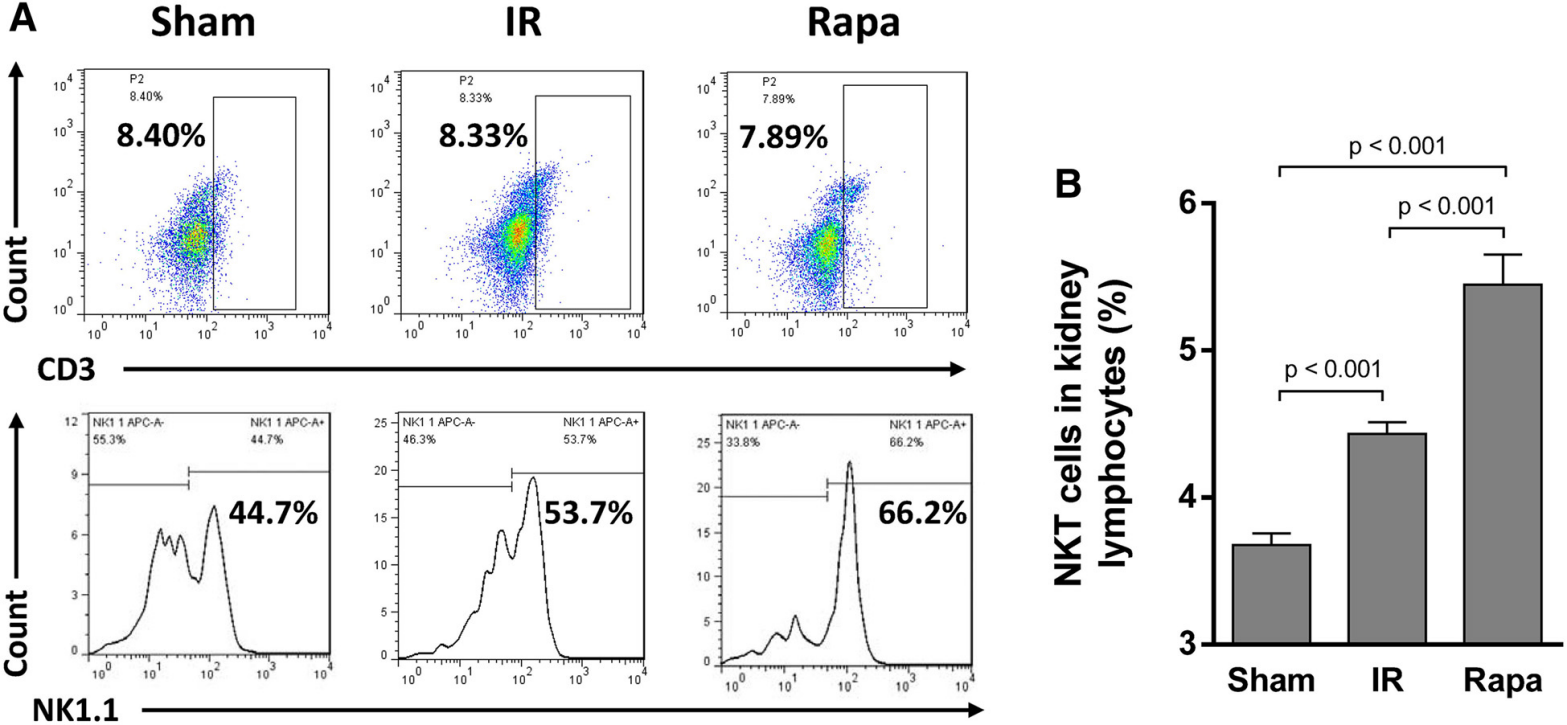
**The proportion of NKT cells in kidney.** Lymphocytes were gated from all RBC-free single cell suspensions by FSC and SSC (not showed), CD3^+^ cells were gated in all lymphocytes, and NK1.1+ cells were selected in CD3^+^ lymphocytes. The proportion of NKT cells (CD3^+^NK1.1^+^) in kidney lymphocytes was calculated as the proportion of CD3^+^ cells in all lymphocytes multiplied by the NK1.1^+^ cells in CD3^+^ lymphocytes **(A)**. The proportion of NKT cells (CD3^+^NK1.1^+^) in kidney tissue was significantly higher in Rapa group than in IR group and Sham group **(B)**. Data are expressed as mean ± SD (n = 5).

To investigate the effect of rapamycin on the trafficking of NKT cells in IR injury, we analyzed the proportions of NKT cells in peripheral blood, spleen and kidney. In spleen, the proportion of NKT cells was decreased in the IR group compared with the sham group, but the difference is not significant. Rapamycin, however, significantly reduced NKT cell proportion (Figure 3). In peripheral blood, the proportion of NKT cells was raised after IR injury. After rapamycin treatment, the proportion of NKT cells was even increased in comparison with the IR group (Figure 4). Although IR injury induced an increase of NKT cells in the injured kidney, rapamycin induced a 5.1 fold increase of NKT cells in the kidney compared with the IR group (Figure 5).

### Rapamycin-induced NKT cell recruitment to the injured kidney was mediated by CXCL9, CXCL10 and CXCR3

**Figure 6:**
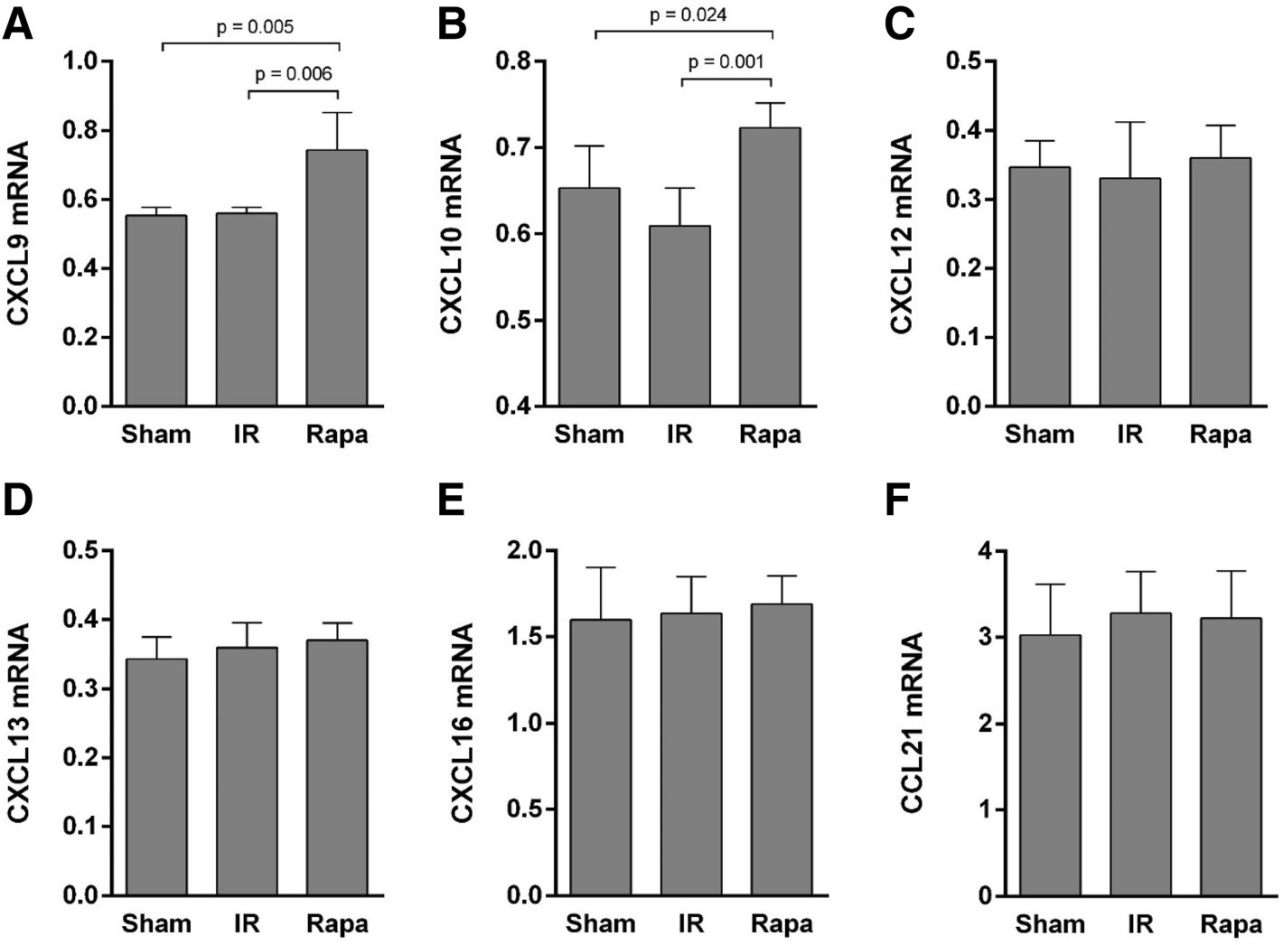
**Expression of chemokines in kidney tissue.** The expression of NKT trafficking-oriented chemokines in kidney were detected by RT-PCR. The expression of CXCL9 and CXCL10 was significantly increased after Rapamycin treatment compared with the IR group **(A-B)**, while the expression of CXCL12, CXCL13, CXCL16 and CCL21 showed no significant difference **(C-F)**. Data are expressed as mean ± SD (n = 5).

**Figure 7:**
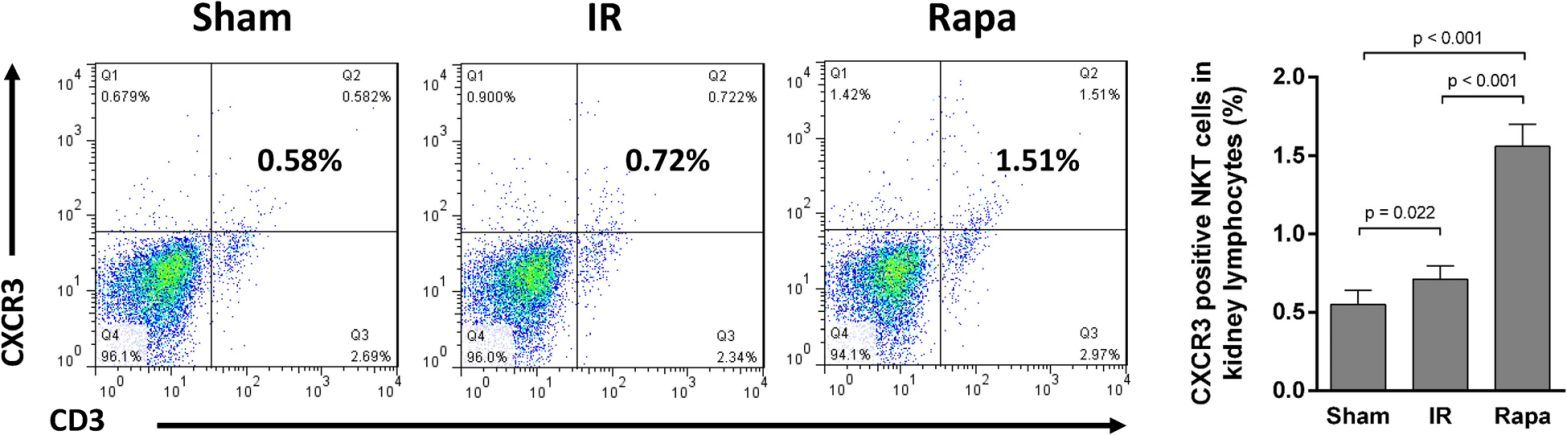
**CXCR3 positive NKT cells in kidney lymphocytes.** After Rapamycin treatment, CXCR3^+^ NKT cells in kidney was significantly elevated compared with IR group and Sham group. Data are expressed as mean ± SD (n = 5).

To verify the chemokines involved in rapamycin-induced recruitment of NKT cells, the expression of a variety of chemokines related to NKT cell trafficking was detected in kidney tissue. The expression of CXCL9 and CXCL10 was significantly increased after rapamycin treatment compared with the IR group (Figure 6A,B), while the expression of CXCL12, CXCL13, CXCL16 and CCL21 showed no significant difference (Figure 6C-F). Correspondingly, the expression of CXCR3, which is the receptor for CXCL9 and CXCL10, was also elevated in rapamycin-treated group compared with the IR group (Figure 7).

## Discussion

In this study, we showed that rapamycin was able to recruit NKT cells to the site of injury in the kidney post 24 h reperfusion. We also demonstrated that this recruitment was directed by chemokines in terms of CXCL9, CXCL10 and their receptor CXCR3. Our research is the first to reveal that rapamycin has an effect on the trafficking process of renoprotective NKT cells.

Rapamycin is a novel kind of immunosuppressive drug widely used in anti-allograft rejection after transplant surgery, but its effect on kidney IR injury still remained controversial. In our preliminary study, we found that rapamycin ameliorated renal IR injury in early stage (1 d after IR injury) while aggravate the injury in late stage (7 d after IR injury), which was associated with its anti-apoptotic and anti-survival effects on different stages of IR injury. In evaluating kidney function 24 h after surgery, this study found that rapamycin treatment significantly reduced Scr and BUN concentrations compared with the IR group, confirming that rapamycin protected kidneys from IR injury in early stage. Accordingly, H&E stained pathological assessment also showed that rapamycin ameliorated tissue damage in the kidneys.

**Figure 8:**
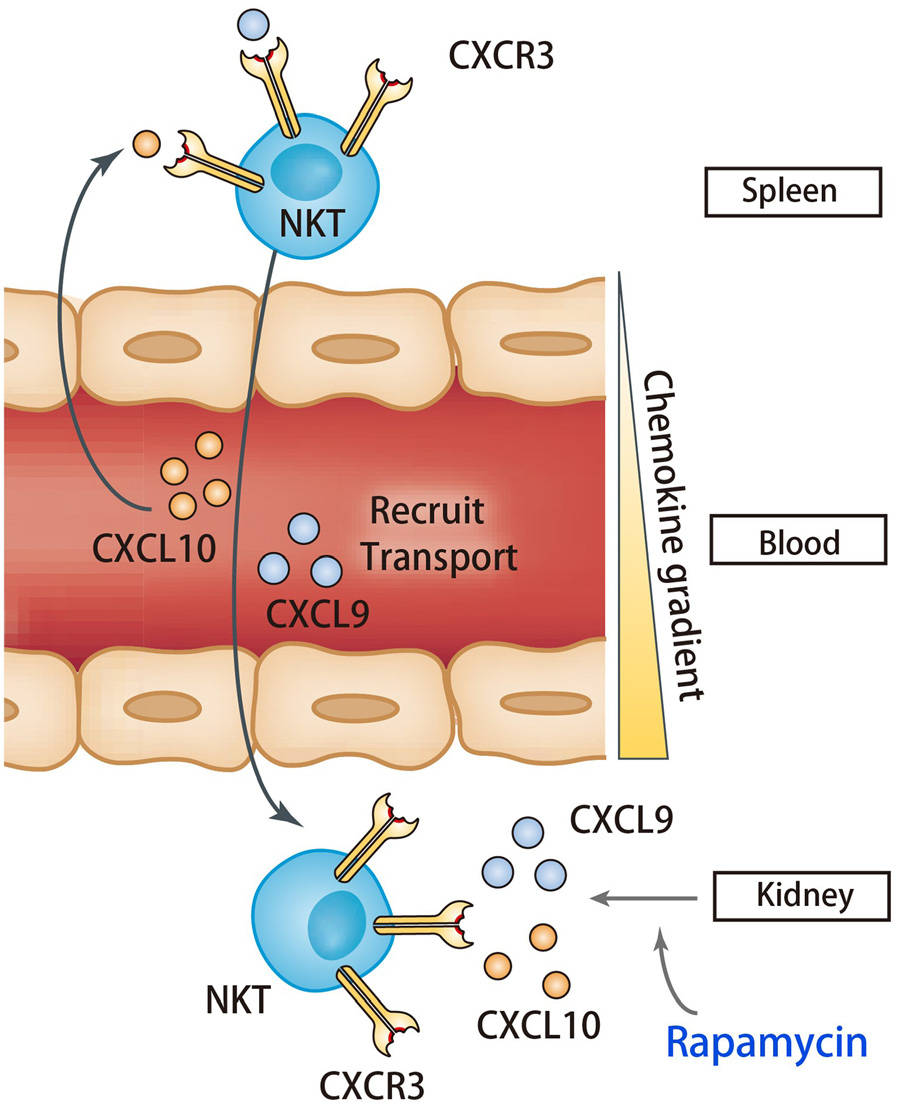
**The schematics of recruitment of NKT cells to kidney after rapamycin treatment.** After rapamycin treatment, chemokine CXCL9 and CXCL10 were highly expressed in kidney to create the gradient of chemokine. The interaction of CXCL9, CXCL10 and their receptor CXCR3 expressed on NKT cells recruited NKT cells from spleen via transport of blood to the targeted site of injury where they played a protective role.

NKT cells, expressing both T cell receptors (TCR) and natural killer cell receptors [18], have been reported to actively participate in ischemia-reperfusion injury in multiple solid organs. Cao et al. found that hepatic preconditioning by preactivation of NKT cells with alpha-GalCer protected the liver from IR injury via an IL-13 and adenosine A2AR-dependent mechanism [19]. In kidney, the study from Li et al. supported the essential role of NKT cells in the innate immune response of renal IR injury by mediating neutrophil infiltration and production of IFN-γ [20]. Consistent with this, Yang et al. reported that sulfatide-reactive NKT cells abrogated renal IR Injury. Modulation of cellular infiltration and cytokine expressions along with molecular changes such as HIF-1α and IL-10 were the main mechanisms for protection [13]. NKT cells were key participants in the early innate response in IR injury and trafficked post-ischemic kidneys as early as 3 h after IR injury [20].Our research showed that in the early stage post reperfusion, the proportion of NKT cells was decreased in spleen and increased in peripheral blood and kidney in rapamycin-treated group in comparison with the IR group. This result suggested that rapamycin recruited NKT cells from spleen where immune cells were stored to the targeted site of injury through transport of peripheral blood. Thus, we ratiocinate upon the facts mentioned above that the renoprotective effect of rapamycin in the early stage probably has close connection with its influence on NKT cell trafficking to the site of injury. Our results demonstrated that the recruitment process was directed by CXCL9, CXCL10 and CXCR3 interaction, while other NKT trafficking-related chemokines like CXCL12, CXCL13, CXCL16 and CCL21 may not be involved in this process. Rapamycin treatment enhanced the expression of CXCL9 and CXCL10 in the IR-induced kidney, thereby creating the chemokine gradient between kidney internal and external tissues. Accordingly, CXCR3^+^ NKT cells in kidney were also increased after rapamycin treatment. Thus, CXCL9 and CXCL10 interacted with the corresponding receptor CXCR3 expressed on NKT cells to recruit NKT cells to IR-induced kidney where they are able to play a protective role (Figure 8). Although the increased CXCL9 and CXCL10 expression was observed in our study, the mechanism that rapamycin increases the expression of CXCL9 and CXCL10 in IR-induced kidney needs to be further investigated, which was the limitation of this study.

## Conclusions

Our study demonstrated that rapamycin was capable of ameliorating renal IR injury in early stage and recruiting NKT cells to IR-induced kidney by CXCL9/10 and CXCR3 interaction. The recruitment of renoprotective NKT cells may account for the protective role of rapamycin. Thus, this study provided an experimental insight for rapamycin as a decent choice for the immunosuppresive regimen in the early stage after renal transplantation. In the future, further investigation aims to reveal the mechanism behind the fact that rapamycin could upregulate the expression certain chemokines to draw NKT cells to IR-induced kidney.

## References

[1] Perico N, Cattaneo D, Sayegh MH, Remuzzi G (2004). Delayed graft function in kidney transplantation. Lancet.

[2] Goncalves GM, Cenedeze MA, Feitoza CQ, de Paula CB, Marques GD, Pinheiro HS, dos Paula Antunes Teixeira V, Antonia dos Reis M, Pacheco-Silva A, Camara NO (2007). The role of immunosuppressive drugs in aggravating renal ischemia and reperfusion injury. Transplant Proc.

[3] Kezic A, Becker JU, Thaiss F (2013). The effect of mTOR-inhibition on NF-kappaB activity in kidney ischemia-reperfusion injury in mice. Transplant Proc.

[4] Goncalves GM, Cenedeze MA, Feitoza CQ, Wang PM, Bertocchi AP, Damiao MJ, Pinheiro HS, Antunes Teixeira VP, dos Reis MA, Pacheco-Silva A, Camara NO (2006). The role of heme oxygenase 1 in rapamycin-induced renal dysfunction after ischemia and reperfusion injury. Kidney Int.

[5] Kezic A, Thaiss F, Becker JU, Tsui TY, Bajcetic M (2013). Effects of everolimus on oxidative stress in kidney model of ischemia/reperfusion injury. Am J Nephrol.

[6] Vogelbacher R, Wittmann S, Braun A, Daniel C, Hugo C (2007). The mTOR inhibitor everolimus induces proteinuria and renal deterioration in the remnant kidney model in the rat. Transplantation.

[7] Castedo M, Ferri KF, Blanco J, Roumier T, Larochette N, Barretina J, Amendola A, Nardacci R, Metivier D, Este JA, Piacentini M, Kroemer G (2001). Human immunodeficiency virus 1 envelope glycoprotein complex-induced apoptosis involves mammalian target of rapamycin/FKBP12-rapamycin-associated protein-mediated p53 phosphorylation. J Exp Med.

[8] Weichhart T, Saemann MD (2009). The multiple facets of mTOR in immunity. Trends Immunol.

[9] Arrenberg P, Halder R, Kumar V (2009). Cross-regulation between distinct natural killer T cell subsets influences immune response to self and foreign antigens. J Cell Physiol.

[10] Johnston B, Kim CH, Soler D, Emoto M, Butcher EC (2003). Differential chemokine responses and homing patterns of murine TCR alpha beta NKT cell subsets. J Immunol.

[11] Yang SH, Jin JZ, Lee SH, Park H, Kim CH, Lee DS, Kim S, Chung NH, Kim YS (2007). Role of NKT cells in allogeneic islet graft survival. Clin Immunol.

[12] Oh K, Kim S, Park SH, Gu H, Roopenian D, Chung DH, Kim YS, Lee DS (2005). Direct regulatory role of NKT cells in allogeneic graft survival is dependent on the quantitative strength of antigenicity. J Immunol.

[13] Yang SH, Lee JP, Jang HR, Cha RH, Han SS, Jeon US, Kim DK, Song J, Lee DS, Kim YS (2011). Sulfatide-reactive natural killer T cells abrogate ischemia-reperfusion injury. J Am Soc Nephrol.

[14] Yang C, Zhao T, Lin M, Zhao Z, Hu L, Jia Y, Xue Y, Xu M, Tang Q, Yang B, Rong R, Zhu T (2013). Helix B surface peptide administered after insult of ischemia reperfusion improved renal function, structure and apoptosis through beta common receptor/erythropoietin receptor and PI3K/Akt pathway in a murine model. Exp Biol Med (Maywood).

[15] Kelly KJ, Williams WW, Colvin RB, Meehan SM, Springer TA, Gutierrez-Ramos JC, Bonventre JV (1996). Intercellular adhesion molecule-1-deficient mice are protected against ischemic renal injury. J Clin Invest.

[16] Yang C, Li L, Xue Y, Zhao Z, Zhao T, Jia Y, Rong R, Xu M, Nicholson ML, Zhu T, Yang B (2013). Innate immunity activation involved in unprotected porcine auto-transplant kidneys preserved by naked caspase-3 siRNA. J Transl Med.

[17] Zhao Z, Yang C, Li L, Zhao T, Wang L, Rong R, Yang B, Xu M, Zhu T (2014). Increased peripheral and local soluble FGL2 in the recovery of renal ischemia reperfusion injury in a porcine kidney auto-transplantation model. J Transl Med.

[18] Taniguchi M, Seino K, Nakayama T (2003). The NKT cell system: bridging innate and acquired immunity. Nat Immunol.

[19] Cao Z, Yuan Y, Jeyabalan G, Du Q, Tsung A, Geller DA, Billiar TR (2009). Preactivation of NKT cells with alpha-GalCer protects against hepatic ischemia-reperfusion injury in mouse by a mechanism involving IL-13 and adenosine A2A receptor. Am J Physiol Gastrointest Liver Physiol.

[20] Li L, Huang L, Sung SS, Lobo PI, Brown MG, Gregg RK, Engelhard VH, Okusa MD (2007). NKT cell activation mediates neutrophil IFN-gamma production and renal ischemia-reperfusion injury. J Immunol.

